# The effect of colchicine on cancer risk in patients with immune-mediated inflammatory diseases: a time-dependent study based on the Taiwan’s National Health Insurance Research Database

**DOI:** 10.1186/s40001-024-01836-1

**Published:** 2024-04-22

**Authors:** Jun-Jun Yeh, Pei-Xuan Liw, Yi-Sin Wong, Husan-Min Kao, Chia-Hsun Lee, Cheng-Li Lin, Chia-Hung Kao

**Affiliations:** 1 Department of Family Medicine, Chest Medicine, Geriatric Medicine and Medical Research, Ditmanson Medical Foundation Chia-Yi Christian Hospital, Chiayi, Taiwan; 2 College of Medicine, China Medical University, Taichung, Taiwan; 3 Department of Family Medicine, Ditmanson Medical Foundation Chia-Yi Christian Hospital, Chiayi, Taiwan; 4 Department of Geriatric Medicine, Ditmanson Medical Foundation Chia-Yi Christian Hospital, Chiayi, Taiwan; 5 Department of Medical Education, Ditmanson Medical Foundation Chia-Yi Christian Hospital, Chiayi, Taiwan; 6 Management Office for Health Data, China Medical University Hospital, Taichung, Taiwan; 7 Graduate Institute of Biomedical Sciences, College of Medicine, China Medical University, No. 2, Yuh-Der Road, Taichung, 404 Taiwan; 8 Department of Nuclear Medicine and PET Center, China Medical University Hospital, Taichung, Taiwan; 9 Department of Bioinformatics and Medical Engineering, Asia University, Taichung, Taiwan; 10 Artificial Intelligence Center, China Medical University Hospital, Taichung, Taiwan

**Keywords:** Colchicine, Cancer risk, Immune-mediated inflammatory diseases

## Abstract

**Background:**

To determine the effect of colchicine on cancer risk in patients with the immune-mediated inflammatory diseases (IMIDs)-related to colchicine use.

**Methods:**

This is a time-dependent propensity-matched general population study based on the National Health Insurance Research Database (NHIRD) of Taiwan. We identified the IMIDs patients (*n* = 111,644) newly diagnosed between 2000 and 2012 based on the International Classification of Diseases, Ninth Revision, Clinical Modification (ICD-9-CM)—274,712, 135, 136.1, 279.49, 518.3, 287.0, 696.0, 696.1, 696.8, 420, 429.4, 710.0, 710.1, 710.3, 710.4, 714.0, 720, 55.0, 55.1, 55.9, 556. Inclusion criteria: aged ≧ 20 years, if a patient had at least these disease diagnosis requirements within 1 year of follow-up, and, these patients had at least two outpatient visits or an inpatient visit. After propensity-matched according to age, sex, comorbidities, medications and index date, the IMIDs patients enter into colchicine users (*N* = 16,026) and colchicine nonusers (*N* = 16,026). Furthermore, time-dependent Cox models were used to analyze cancer risk in propensity-matched colchicine users compared with the nonusers. The cumulative cancer incidence was analyzed using Cox proportional regression analysis. We calculated adjusted hazard ratios (aHRs) and their 95% confidence intervals (95% CIs) for cancer after adjusting for sex, age, comorbidities, and use of medicine including acetylcysteine, medication for smoking cessation such as nicotine replacement medicines (the nicotine patch) and pill medicines (varenicline), anti-inflammatory drugs and immunosuppressant drugs.

**Results:**

Comparing the colchicine nonusers, all cancer risk were mildly attenuated, the (aHR (95% CI)) of all cancer is (0.84 (0.55, 0.99)). Meanwhile, the colchicine users were associated with the lower incidence of the colorectal cancer, the (aHRs (95% CI)) is (0.22 (0.19, 0.89)). Those aged < 65 years and male/female having the colchicine users were associated with lower risk the colorectal cancer also. Moreover, the colchicine > 20 days use with the lower aHR for colorectal cancer.

**Conclusion:**

Colchicine was associated with the lower aHR of the all cancer and colorectal cancer formation in patients with the IMIDs.

**Supplementary Information:**

The online version contains supplementary material available at 10.1186/s40001-024-01836-1.

## Introduction

The pyrin domain-containing protein 3 [or NOD-like receptor protein 3 (NLRP3)] inflammasome and NLRP3 inflammasome have been associated with colorectal cancer formation [1]. Notably, these markers would increase, especially in patients with the gout, diabetes, obesity and cancer [1]. The role of the NLRP3 inflammasome varies according to sex, age, and tumor type. For example, estrogen enhances the expression of NLRP3 inflammasome, whereas testosterone inhibits its expression. The estrogen acts through estrogen receptor β (ERβ) to enhance the activation of NLPR3 inflammasome and promote the progression of endometrial cancer [2]. In contrast, NLRP3 inflammasome protects against the bowel inflammation associated-tumor formation in chronic stages but promotes colon cancer in the early stage of the chronic colitis-ulcerative colitis, Crohn’s disease [1, 3, 4]. Thus, the NLRP3 inflammasome may be critically involved in the maintenance of intestinal homeostasis and protection against colitis [4]. Hence, NLRP3 inflammasome exhibits contrasting roles in cancer development [1, 5].

Colchicine is an anti-inflammatory drug, it have been used in the gout and immune-mediated inflammatory diseases (IMIDs) such as arthritis-**c**rystal arthropathies, systemic inflammatory diseases such as sarcoidosis, Behcet’s syndrome, autoimmune disease, chronic idiopathic or spontaneous urticarial skin diseases, allergic purpura, psoriasis, collagen vascular diseases, and pericarditis [3, 6–9]. In addition, colchicine exerts its unique effects by the retardation of new tubulin formation, dissociation of tubulin, thus leading to cancer cell death and it could inhibit angiogenesis and cancer cell migration and metastasis, limit ATP influx into mitochondria, and release caspases and cytochrome-c, thus leading to apoptosis [1, 10]. In recent study, the colchicine could reduce the NLRP3 inflammasome activity or its downstream mediators in the cancer related to chronic inflammation diseases such as the hyperlipidemia with atherosclerosis, tobacco use with chronic obstructive pulmonary disease (COPD) and chronic colitis [1, 5, 11–13]. The nano-drug delivery of colchicine by means of mesoporous silica nanoparticles could enhance the anticancer effect for attenuating the risk of colon cancer cell formation support these speculations [14].

The important evidence that links inflammation and cancer is that non-steroidal anti-inflammatory drugs (NSAIDs), such as aspirin, reduce the risk and mortality from many cancers [15]. Kuo et al. showed that male gout patients using colchicine had less colorectal cancer than other gout patients and colchicine use was associated with a decreased risk of incident all-cause cancers [16]. The IMIDs such as gout, chronic colitis are associated with the risk of cancer [9, 17–19]. However, to best our knowledge in English literature, no research has examined the role of colchicine in the evolution of cancer among patients with IMIDs. Thus, in this study, we investigated the relationship between the effects of colchicine and the incident cancer in patients with IMIDs by using a National Health Insurance Research Database (NHIRD).

## Methods

### Data source

On March 1, 1995, Taiwan launched a single-payer mandatory enrollment National Health Insurance (NHI) Program. Taiwan’s National Health Research Institutes (NHRI) established from 2002. The NHRI continue to maintain NHIRD for the purpose of public research. Up to 99.99% of Taiwan’s population are enrolled under this well-established program. Thus, the NHIRD, derived from claims data of NHI beneficiaries, could illuminate the disease burden and health care process of the entire Taiwanese population and the population included in this database representative of the population of Taiwan.

We analyzed the Longitudinal Health Insurance Database, a subset of the NHIRD that contains demographic information, inpatient and outpatient records, medications, and treatments of 2 million insured individuals.

### Study population

Between the 2000-01-01 and the 2012-12-31, the patients with the ICD-9CM (274, 712, 135, 136.1, 279.49, 518.3, 287.0, 696.0, 696.1, 696.8, 420, 429.4, 710.0, 710.1, 710.3, 710.4, 714.0, 720, 55.0, 55.1, 55.9, 556)-IDIMs enter into study. The inclusion criteria (*N* = 111,644) as below: aged ≧ 20 years, if a patient had at least these disease diagnosis requirements within 1 year of follow-up. And, these patients had at least two outpatient visits or an inpatient visit.

The patients who used colchicine between 2000 and 2012 were defined as colchicine users among patients with IMIDs (*N* = 23,612) [6–8, 20, 21]. The colchicine users need to fulfill criteria as below: in Lin et al.’s study, the nude mice experiment showed that colchicine-treated mice after 14 days of treatment had lower increased tumor volume ratios and tumor growth rates than the control. In accordance with this previous study, we set the duration of the colchicine use > 15 days [22]. Nonusers defined as those who never used colchicine or the date of the first prescription of colchicine, the duration of the colchicine use ≤ 14 days (*N* = 88,032) [15, 23] (Additional file 1: Figure S1).

Excluding criteria (*N* = 1148) in colchicine users as below: not Taiwanese citizens, or aged < 20 years, had no IMIDs-related prescription requirements or related management or procedure 1 year after the first IMIDs diagnosis; had a disease history of IMIDs or received colchicine before the first IMIDs diagnosis or previous history of cancer (*ICD-9-CM* codes 140–239) [7, 21, 24]. The colchicine may be considered as an immunosuppressant agent, and the colchicine may interact with other immunosuppressant. And these immunosuppressants could interact with each other and perhaps affecting the cancer formation [25, 26]. For those who had more than one prescription of immunosuppressant, a minimum of < 7 days between prescriptions were excluded. Incident use of drugs increases in the months prior to a cancer diagnosis. To avoid reverse causation, 6 months lag time would be sufficient for most drug–cancer associations. In this study, to avoid this reverse causation, the lag time would be set as ≧ 12 months from the first prescription of colchicine to any diagnosis of cancer [23, 27]. Therefore, prescriptions within 12 months of diagnosis of cancer were not considered as exposed, again to avoid reverse causation [25]. And the same exclusion criteria was used in the nonusers.

Before propensity matching, 22,464 cases without cancer at baseline in colchicine users and 76,224 controls were selected (*n* = 98,688). The 1:1 propensity score matching was used in cohort studies And, we excluded unmatched 66,636 patients. Finally, total 16,026 colchicine used cases and 16,026 control subjects were identified in the study cohort (Additional file 1: Figure S1).

Since this a time-dependent study, the index date for the colchicine users was the date of the first prescription of colchicine, and a date between 2000 and 2012 was the index date for the nonusers. Colchicine users and nonusers were then matched by propensity scores according to age, sex, comorbidities (alcohol-related disease, coronary artery disease, diabetes, hypertension, hyperlipidemia, chronic obstructive pulmonary disease stroke, tobacco use, depression, chronic kidney disease), medications (acetylcysteine, smoking cessation-related drugs, anti-inflammatory drugs and immunosuppressants) and index date. The *International Classification of Diseases, Ninth Revision, Clinical Modification* (*ICD-9-CM*) diagnosis codes considered for patients with the MD (Full names, Additional file 2: Table S1).

### Licensing Committee (ethics statement)

The NHIRD encrypts patient personal information to protect privacy and provides researchers with anonymous identification numbers associated with relevant claims information, including sex, date of birth, medical services received, and prescriptions. All methods were carried out in accordance with relevant guidelines and regulations. Therefore, patient consent is not required to access the NHIRD. This study was approved to fulfill the condition for exemption by the Institutional Review Board (IRB) of China Medical University & Hospital Research Ethics Center (CMUH104-REC2-115[CR-7]). The IRB also specifically waived the consent requirement.

### Data availability statement

The dataset used in this study is held by the Taiwan Ministry of Health and Welfare (MOHW). The Ministry of Health and Welfare must approve our application to access this data. Any researcher interested in accessing this dataset can submit an application form to the Ministry of Health and Welfare requesting access. Please contact the staff of MOHW (Email: stcarolwu@mohw.gov.tw) for further assistance. Taiwan Ministry of Health and Welfare Address: No.488, Sec. 6, Zhongxiao E. Rd., Nangang Dist., Taipei City 115, Taiwan (R.O.C.). Phone: +886-2-8590-6848. All relevant data are within the paper.

### Main outcome and covariates

The primary endpoint of this study was the development of cancer (*ICD-9-CM* codes 140–239), as evidenced by a major illness or injury certificate of cancer. The study endpoint was defined as the occurrence of cancer, withdrawal from the insurance program if they are missing for 6 months or more or an individual is missing because of a natural disaster, coverage can be withdrawn from the day the disaster occurred or they lose Taiwan citizenship, move overseas, or have an expired Alien Resident Certificate, or December 31, 2013.

In addition to age and sex, other potential confounders included comorbidities (Full names, Additional file 2: Table S1). The use of acetylcysteine, smoking cessation-related medication, anti-inflammatory drugs (aspirin, NSAID) and immunosuppressant drugs was evaluated as a confounder also. The severity and duration of IMIDs have impact on the risk of cancer [28–30]. Owing to the comorbidities and medications were close relation to the IMIDs severity and duration [28]. Thus, these comorbidities, medications and Charlson comorbidity index enter into analysis in this study. Meanwhile, we used alcohol-related diseases as a proxy for drinking, chronic obstructive pulmonary disease (COPD) and tobacco use for smoking. The Anatomical Therapeutic Chemical code (ATC) of medications were defined as a prescription with one of the following National Health Insurance (NHI) codes: Additional file 1: Figure S1 displays the flowchart for selection of the patients.

### Statistical analysis

Categorical variables were expressed as number and percentage, and differences between the two cohorts were examined using the Chi-square test. Continuous variables were presented as median and interquartile range, and differences between the two cohorts were assessed using the Mann–Whitney *U* test. The incidence rate was expressed as per 1000 person-years. The disease duration might impact on the risk of cancer and should be taken into account. Thus, the Cox proportional hazards (PH) model with time-dependent exposure covariates was used as the main model in the study to analyze the effect of colchicine on cancer. The association between the duration of colchicine use and the development of cancer was analyzed using the general Cox PH model. The Kaplan–Meier method was used to obtain the cumulative curves, and the results were then examined using the log-rank test. SAS statistical software (version 9.1, SAS Institute, Cary, NC) was used to perform the analysis. A *p*-value less than 0.05 was set as the significant level.

## Results

A total of 22,464 patients were defined as colchicine users in this study (Table 1). The median age of the colchicine users was higher than that of the colchicine nonusers: 54.3 years versus 53.7 years, respectively. A male preponderance was observed in the colchicine users compared with the colchicine nonusers. As this is a time-dependent study, more patients had developed comorbidities such as alcohol-related diseases, diabetes, hypertension and hyperlipidemia in the colchicine users than in the colchicine nonusers. In addition, the colchicine users included more patients who used acetylcysteine, smoking cessation-related medication, anti-inflammatory drugs such as oral steroids (OSs), NSAIDs, acetylsalicylic acid, and immunosuppressant drugs such as azathioprine, sulfasalazine, cyclophosphamide, methotrexate, hydroxychloroquine, and cyclosporine. After propensity score matching, both cohorts included 16,026 patients each. Both cohorts had a similar distribution of age, comorbidities, and medications except the sex and alcohol-related disease, hypertension and hyperlipidemia.

**Table 1:** Demographic characteristics and comorbidities in the propensity-score-matched cohorts with and without colchicine used among patients with the immune-mediated inflammatory diseases

Variable	Not matched		*p*-value	Propensity score matched		*p*-value
	Colchicine			Colchicine		
	No	Yes		No	Yes	
	*N* = 76,224	N = 22,464		*N* = 16,026	N = 16,026	
Age, year			< 0.001			0.06
≤ 49	37,581 (49.3)	8818 (39.3)		6073 (37.9)	6279 (39.2)	
50–64	24,818 (32.6)	7828 (34.9)		6009 (37.5)	5870 (36.6)	
65+	13,825 (18.1)	5818 (25.9)		3944 (24.6)	3877 (24.2)	
Median ± (IQR)^a^	50.3 (38.5–60.8)	54.0 (43.8–65.4)		53.7 (43.7–64.6)	54.3 (44.1–64.8)	
Sex			< 0.001			0.001*
Female	44,370 (58.2)	7884 (35.1)		7700 (48.1)	7138 (44.5)	
Male	31,854 (41.8)	14,580 (64.9)		8326 (52.0)	8888 (55.5)	
Comorbidities						
Alcohol-related illness	30,956 (40.6)	11,856 (52.8)	< 0.001	8364 (52.2)	8550 (53.4)	0.04*
CAD	1860 (2.44)	1136 (5.06)	< 0.001	575 (3.59)	597 (3.73)	0.51
Diabetes	11,059 (14.5)	4860 (21.6)	< 0.001	3138 (19.6)	3285 (20.5)	0.04*
Hypertension	40,059 (52.6)	16,264 (72.4)	< 0.001	10,965 (68.4)	11,157 (69.6)	0.02*
Hyperlipidemia	49,968 (65.6)	18,081 (80.5)	< 0.001	12,942 (80.8)	12,784 (79.8)	0.03*
COPD	5366 (7.04)	3872 (17.2)	< 0.001	2020 (12.6)	2016 (12.6)	0.95
Stroke	5878 (7.71)	2795 (12.4)	< 0.001	1731 (10.8)	1684 (10.5)	0.39
Depression	10,193 (13.4)	15,754 (70.1)	< 0.001	9318 (58.1)	9316 (58.1)	0.98
Tobacco use	5878 (7.71)	2795 (12.4)	< 0.001	1856 (11.6)	1874 (11.7)	0.75
Chronic kidney disease	5258 (6.90)	2365 (10.5)	< 0.001	1544 (9.63)	1517 (9.47)	0.61
Charlson comorbidity index ≧1	26,500 (34.6)	11,400 (50.6)	< 0.001	7600 (47.4)	7578 (47.2)	0.96
Medications						
Acetylcysteine	7914 (10.4)	3034 (13.5)	< 0.001	2066 (12.9)	2045 (12.8)	0.73
Smoking cessation-related	3611 (4.74)	1598 (7.11)	< 0.001	982 (6.13)	938 (5.85)	0.30
Anti-inflammatory and immunosuppressant drugs	34,654 (45.5)	14,619 (65.1)	< 0.001	9192 (57.4)	9691 (60.5)	0.01*

*CAD* coronary artery disease, *COPD* chronic obstructive pulmonary disease

Smoking cessation-related: nicotine replacement medicines (the nicotine patch) and pill medicines (varenicline)

Anti-inflammatory and immunosuppressant drugs: oral steroids,aspirin, non-steroidal anti-inflammatory drugs, acetylsalicylic acid, azathioprine, sulfasalazine, cyclophosphamide, methotrexate, hydroxychloroquine, cyclosporine

Chi-square test; ^a^Mann–Whitney test

^*^p < 0.05

However, Charlson comorbidity index (CCI) have been found to be strongly predictive of survival among colon cancer patients [29]. The CCI index ≧ 1 of the both cohorts was without significant differences [31]. Furthermore, we use the multiple variants logic regression with time-dependent analysis for minimize these baseline imbalance [32].

The higher frequency of the gout in colchicine users was in parallel with the higher frequency of alcohol-related disease, hypertension and hyperlipidemia. In our study, the 75% colchicine users (*n* = 16,026 × 0.75 = 12,019) having the gout (Additional file 2: Table S1). The number of the colchicine users with gout is 12,019 which is similar to the Kuo et al. study (a total of 13,679 patients with gout having received colchicine).

**Fig. 1:**
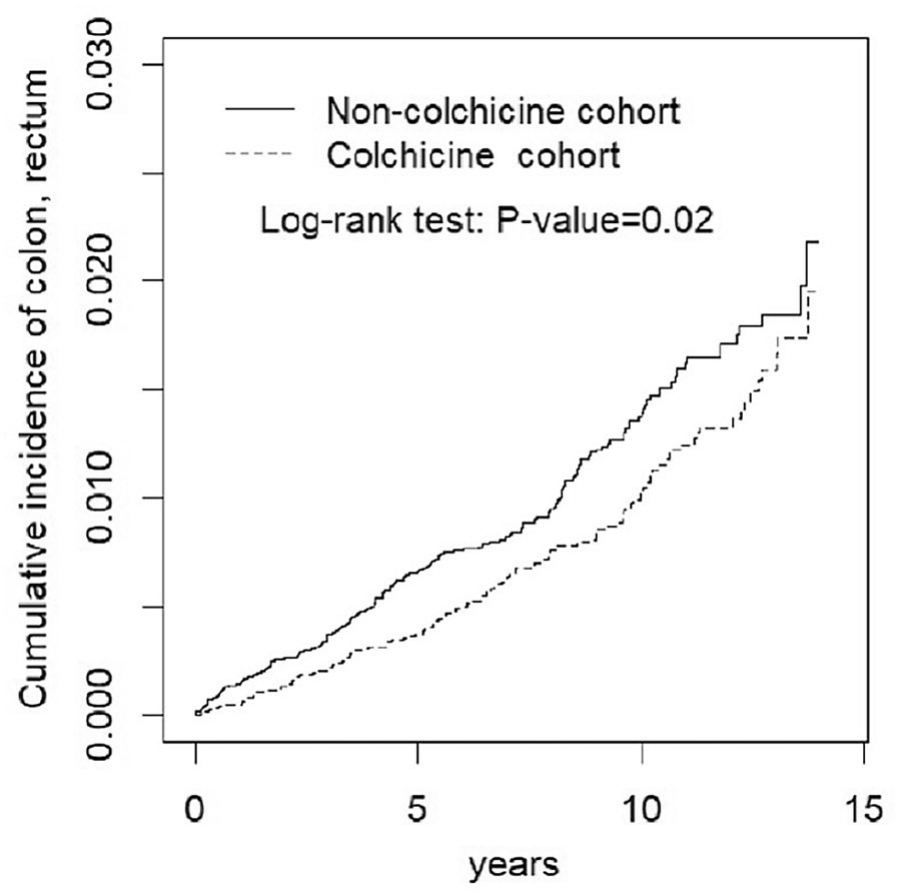
Cumulative incidence of colorectal cancer curves for colchicine users and colchicine nonusers by propensity score matched

Table 2 illustrates the association of colchicine use with different types of cancer in the colchicine users. For example, comparing with colchicine nonusers, the colorectal cancer and brain tumor in the colchicine users were associated with the different adjusted HRs [aHRs], the aHRs (95% CI) were 0.22 (0.19, 0.89), and 0.68 (0.42, 2.01), respectively. The colchicine users were associated with the lower incidence of the colorectal cancer **(**Fig. 1**).** Additional file 3: Table S2 displays the lower aHR for all cancer revealed in the colchicine users.

**Table 2:** Incidence of individual cancer (per 1000 person-years) and estimated hazard ratios in the immune-mediated inflammatory diseases with colchicine compared to the immune-mediated inflammatory diseases without colchicine by Cox proportional hazard model with time-dependent covariates in propensity score-matched cohorts

Site of cancers	Colchicine				Crude HR (95% CI)	Adjusted HR^b^ (95% CI)
	No (*N* = 16,026)		Yes (*N* = 16,026)			
	Event	Rate^a^	Event	Rate^a^		
Hematologic malignancy	55	0.44	40	0.33	1.33 (0.60, 2.17)	1.10 (0.58, 2.14)
Head and neck cancer	49	0.40	43	0.48	1.20 (0.92, 3.44)	1.21 (0.85, 3.03)
Esophagus	23	0.19	18	0.15	0.78 (0.53, 3.55)	1.00 (0.52, 3.63)
Stomach	50	0.40	41	0.43	1.05 (0.96, 3.97)	1.17 (0.92, 3.77)
Colorectal cancer	170	1.37	39	0.27	0.20 (0.12, 0.97)*	0.22 (0.19, 0.89)*
Liver	123	0.99	109	0.89	0.90 (0.78, 2.20)	1.00 (0.85, 1.92)
Pancreas	21	0.17	16	0.13	0.76 (0.43, 5.12)	0.89 (0.74, 4.70)
Lung	120	0.97	102	0.83	0.85 (0.80, 1.91)	0.83 (0.76, 1.82)
Skin	19	0.15	17	0.14	0.93 (0.56, 4.09)	0.74 (0.50, 4.30)
Breast cancer	70	1.13	56	0.98	0.87 (0.65, 2.05)	0.86 (0.66, 2.67)
Immune-related cancers	174	1.41	141	1.15	0.82 (0.79, 1.69)	0.83 (0.71, 1.52)
Cervix	17	0.28	16	0.28	–	–
Endometrium	23	0.37	10	0.18	–	–
Ovary	11	0.18	4	0.07	0.39 (0.18, 9.2)	0.42 (0.21, 10.1)
Prostate	67	1.08	48	0.73	0.68 (0.50, 2.05)	0.87 (0.67, 2.01)
Bladder, kidney	72	0.58	63	0.51	0.88 (0.55, 2.87)	0.99 (0.90, 2.64)
Brain	4	0.03	2	0.02	0.66 (0.58,2.00)	0.68 (0.42, 2.01)
Thyroid	21	0.17	16	0.13	0.76 (0.15, 2.56)	0.80 (0.20, 3.56)
Others	43	0.35	32	0.26	0.74 (0.37, 2.03)	0.81 (0.35, 1.99)

Rate^a^, incidence rate, per 1000 person-years; Crude HR, relative hazard ratio

^b^Adjusting for age, sex, comorbidities and medications;

^*^p < 0.05, **p < 0.01

Table 3 illustrates the colchicine use with > 20 days having the lower aHR for the chronic colitis related to colorectal cancer.

**Table 3:** Incidence and adjusted hazard ratio of colorectal cancer stratified by duration of colchicine therapy in patients with the immune-mediated inflammatory diseases in the propensity score-matched cohort

Medication exposed	*N*	Event	Person-year	Rate	Adjusted HR (95% CI)^b^
Colorectal cancer					
Colchicine^a^					
Non-colchicine	16,026	170	123,693	1.37	1.00
> 15 ≤ 20 days	8099	18	61,764	1.10	0.81 (0.61, 1.08)
21–100 days	4036	13	30,949	0.80	0.58 (0.45, 0.99)*
> 100 days	3891	8	30,096	0.23	0.17 (0.15, 0.66)**

^a^The cumulative use day are partitioned in to 3 segments by median, and third quartile

Adjusted HR^b^: multivariable analysis including age, sex, comorbidities and medications

^*^p < 0.05, **p < 0.01

### Investigating the impact of sex and age on the IMIDs and subcohort of IMIDs-chronic colitis-related to colorectal cancers formation

The elderly with colchicine use having the null effect for the aHR of colorectal cancer. Regarding the young adults (aged < 65 years and male/female), the aHRs for colorectal cancer were associated with the lower aHRs for the colchicine users in the IMIDs (Table 4).

**Table 4:** Incidence and hazards ratio of colorectal cancer measured by age, sex, comorbidities and medications in the propensity score-matched cohorts by Cox proportional hazard models with time-dependent exposure covariates among the immune-mediated inflammatory diseases

Variables	Propensity score matched				
	Colchicine				Adjusted HR^b^ (95% CI)
	No (*N* = 16,026)		Yes (*N* = 16,026)		
	Event	Rate^a^	Event	Rate^a^	
Colorectal cancer					
Age, years					
≤ 49	22	0.43	9	0.17	0.27(0.12,0.83)**
50–64	82	1.78	18	0.47	0.79(0.40, 0.59)**
65+	66	2.49	12	0.75	0.88(0.36, 3.49)
Sex					
Female	71	1.15	23	0.56	0.47(0.31, 0.66)**
Male	99	1.60	16	0.79	0.61(0.55, 0.89)*

Rate^a^, incidence rate, per 1000 person-years; adjusted HR^b^: multivariable analysis including age, sex, comorbidities and medications

^*^p < 0.05; **p < 0.01

### The subcohort of the IDIMs-chronic colitis is well representative of these speculations. We display these points in Additional file 4: Table S3

Additional file 4: Table S3 displays the lower aHR of colorectal cancer for the colchicine users among the chronic colitis-(ulcerative colitis, Crohn’s disease) in young adult [9, 18, 33].

### Covariates as proxies to the NLRP3 expression for the incident cancer

The NLRP3 expression were unavailable in the NHIRD. However, the NLRP3 expression was in accordance with the cell type, duration of use, age, sex. Meanwhile, these variables were independent and critical factors of the colchicine on the cancer promotion [22, 34–37]. Moreover, those covariates such as comorbidities (e.g., alcohol-related disease, hypertension, diabetes, depression, tobacco use) were proxies to the NLRP3 expression for the incident cancer [35, 38]. Therein, these variants were entered into the analysis [1, 39].

Additional file 5: Table S4a and Additional file 6: Table S4b display the aHR for the individual comorbidity and medication. The comorbidities display the higher aHR for all cancer except the hypertension and coronary artery disease. The smoking cessation-related medications and anti-inflammatory medications revealed the lower aHR for all cancer.

### Sensitivity analysis-propensity score analysis for time-dependent exposure in different cancer subtype and comorbidities

The colchicine users have the null effect for the aHR of most of the individual cancer such as the brain tumor except the lower aHR of colorectal cancer. Combination of the NLRP3 dual effect for cancer, colchicine modulates NLRP3 effect for cancer, duration of drug effect and detection bias could play a role for contributing to these results. We did sensitivity analysis for clarifying these points for colorectal cancer. We displayed these speculations in Table 5: (1) the combination effect of the colchicine, NLRP3, comorbidities and detection bias on the risk of cancer in colorectal cancer with chronic colitis; (2) sex and age for modulating the effect of colchicine on the risk of colorectal cancer [40, 41].

**Table 5:** Summary findings and speculations

Without stratification with age and sex					
NLRP3	1 NLRP3 dual effect for cancer	2 Colchicine modulate NLRP3 effect for cancer	3 Hyperlipidemia Hypertension Diabetes Liver cirrhosis	4 Detection Bias Modulate aHR^b,c^	aHR^a^
					Effect of 1 + 2 + 3 + 4 =
Colorectal cancer—IMIDs colitis (− attenuating tumorigenic activity late stage of colitis + enhancing tumorigenic activity in early stage of colitis)	±	−−	+	0/+	–

Stratification with age and sex					
	1 NLRP3 effect for cancer	2 Testosterone (+) Estrogen (?) Effect For cancer	3 Hyperlipidemia Hypertension Diabetes Tobacco use	4 Colchicine Modulate NLRP3 Effect for cancer	aHR^a^ Effect of 1 + 2 + 3 + 4 =
Colorectal cancer^c^					
Male	+	+	+	−−	–
Female	+	?	+	−−	–
> 65-year	+	0	+	0/−	0
< 65-year	+	+	+	−−	–

+ were associated with enhancing the + aHR, -were associated with attenuating-aHR, 0 were null effect on the aHR

aHR^a^ effect of 1 + 2 + 3 + 4

*IMIDs* immune-mediated inflammatory diseases

Chronic colitis: the ulcerative colitis, Crohn’s disease

^b^The colchicine users have received the higher frequency of medical services (*N* ≧ 4) with the at least one procedure in relation to cancer diagnosis on these cancers than the colchicine nonusers (*N* = 1)

^c^ In colchicine users, the frequency of high up to 43.2% patients in brain cancer cohort have received the brain-related procedure for neurodegenerative diseases or head trauma. In contrast, in colorectal cancer cohort, only13% patients have received the colonscopy-related procedure. However, these colchicine users having the higher frequency of the examination of the cancers-related procedures than the colchicine nonusers before the diagnosis of these cancers

## Discussion

The main finding of our study was that colchicine use was associated with a lower risk of the colorectal cancer in patients with the IMIDs. The Kaplan–Meier was used only one variable (event of interest-colchicine use) for estimating the survival curves. Log-rank test compares two or more survival curves and does not consider additional independent variables. Similar to multiple regression model, Cox PH model considers additional independent variables (covariates-age, sex, comorbidities and medications) for estimating the differences between the survival curves. To the best of our knowledge, this study is the first study to report an association between colchicine use and incidental cancers in patients with the IMIDs, who were associated with the lower risk of colorectal cancer based on the Cox PH model with time-dependent exposure. Similar to that, the colchicine users were associated with the lower cumulative incidence of the colorectal cancer in analysis of the Kaplan–Meier method. Meanwhile, the lower aHR for all cancer was revealed in the colchicine users also.

The methods of this study are different from the Kuo et al. study. The Kuo et al.’s study focusses on the gout cohort, ever user (colchicine usage within 3 years after gout) and they excluded the DM and cancer within 1 year after gout. In our study, the study cohort is the IMIDs cohort, the patients with colchicine use are all new colchicine users and the colchicine users excluding the lag time within 1 year for incident cancer formation. However, some results are similar to Kuo et al. study. For example, Kuo et al. found that colchicine ever-users had significantly lower incidence of all-cause cancers after adjustment for age, compared with colchicine never-users (aHR = 0.85, 95% CI (0.77, 0.94)). In our study, the lower risk of the all cancer (aHR = 0.84, 95% CI (0.55, 0.99)) was found in the colchicine users excluding the lag time within 1 year for incident cancer. In Kuo et al., the comorbidity such as the hyperlipidemia has higher risk of cancer, on comparing the colchicine ever-users with the colchicine never-users. Similar to that, in our study, the colchicine users with comorbidities such as the alcohol-related disease, diabetes, hyperlipidemia, COPD, depression, tobacco use and chronic kidney disease had higher aHR for cancer.

An important confounding factor of this study is the detection bias. Detection bias is hypothesized, in part, because of observed differences between the colchicine users and colchicine nonusers. Detection bias occurs when screening and diagnostic patterns vary in association with potential risk factors. Detection bias could exaggerate or attenuate estimated cancer–colchicine associations and affecting the aHR for cancer. For example, those aged > 65 received the higher frequency of neurodegenerative diseases-related procedure and brain CT, these procedures let us to early detection of brain lesion. In similar indication, brain CT is a popular examination for resolving the critical neurological patients at emergency department in Taiwan. These feasible brain CT examinations in practice, increase the early detection of the brain tumor [42].

Moreover, the colchicine users have the higher frequency of the alcohol-related diseases, hypertension and hyperlipidemia. The alcohol-related diseases, diabetes, hyperlipidemia have the higher risk of the cancer in our study. Bao et al. report that high glucose promotes human glioblastoma cell growth by increasing the expression and function of chemoattractant and growth factor receptors, leading to the growth of human glioblastoma cells [43]. Rogers et al. report that hyperlipidemia and diabetes are associated with the higher risk of brain tumor such as glioblastoma [44]. These previous reports support these findings. These comorbidities combined with the overactivity of NLRP3 inflammasome attenuate the auxiliary anti-tumor effect of colchicine in patients with IMIDs [37, 45]. Therefore, these combination factors (age, comorbidities and detection bias) lead to null effect for aHR of brain tumor in the IMIDs with colchicine user [46].

Another important finding in this report is that the young adult colchicine users were associated with the lower aHR of colorectal cancer among the IMIDs [21, 47].

As mention before, the NLRP3 inflammasome could play a positive role for protecting the colorectal cancer (negative effect on the aHR) in the late stage of chronic colitis. In the young male with high level of the NLRP3 inflammasome activity especially the IMIDs-chronic colitis patients, testosterone could modulate the NLRP3 inflammasome activity and promoting the NLRP3 inflammasome-related tumor genesis activity [37]. However, colchicine could impair testosterone synthesis, attenuating the effect the testosterone on the NLRP3 inflammasome-related tumor genesis activity, leading to colchicine may play a positive role for protecting the cancer formation (negative effect on the aHR) [48]. Taking these together, the colchicine users were associated with the optimal counterbalance effect on the NLRP3 inflammasome, thus the young male patients in the colchicine users were associated with the lower risk of the colorectal cancer (negative effect on the aHR) in a later course of the chronic colitis even with the higher frequency of the comorbidities (Tables 4, 5) [48, 49]. Similar to that, the colchicine could modulate the NLRP3 inflammasome among the female, leading to colchicine users being associated with the lower aHR for colorectal cancer [35].

The chronic colitis is a key factor of the colorectal cancer. In previous study, the colchicine may play an auxiliary for management of the chronic colitis such as Crohn’s disease with amyloidosis in young adult, perhaps leading to the lower risk of the colorectal cancer in accordance with our findings [3, 50, 51].

In summary, age, sex, comorbidities and detection bias are confounding factors for the effect of colchicine on cancer. However, the colchicine users having the lower risk of the all cancer. Furthermore, especially in young adults and long-term use (> 100 days) having the lowest the aHR for colorectal cancer among the IMIDs.

Finally, subcohort such as the IMIDs-chronic colitis in young adult, those with colchicine use having the lower risk of the colorectal cancer in accordance with initial results.

Altogether, colchicine users are more likely to engage in health-seeking behaviors than colchicine nonusers. Meanwhile, those colchicine users who often use other medications such as anti-hypertension, statins and oral sugar or insulin sometimes forego preventive services such as cancer screening. Differential screening rates by colchicine use could then inflate the aHR for the tumor such as the brain tumor. It is to be noted that when we interpret these findings, we need to take these confounding factors into account [42, 52]. Further research is warranted to confirm these observations.

### Clinical implication in practice

The microtubule-destabilizing agents bind to the colchicine-binding site (CBS) of tubulin, termed colchicine-binding site inhibitors (CBSIs). While colchicine itself could not be used in the treatment of cancer due to systemic toxicity, several CBSIs show promise in the future treatment of different subtypes cancers such as gastro-entero-pancreatic neuroendocrine cancers, including those with acquired multiple drug resistance characteristics [53].

Recently, Hamid et al. report that out of the 69,260,780 patients in the database, they identified 209, 020 patients with ulcerative colitis (0.30%) of whom 9130 had gout (4.3%). Additionally, 249,480 had Crohn’s disease (0.36%) of whom 14,000 had gout (5.61%) [33], such that, the number of the gout with IMIDs-chronic colitis is 9130 + 14,000 = 23,130. We assumed the 75% gout patients with colchicine use, the number of colchicine user is about (23,130) × 0.75 = 17,348. This number of colchicine users is similar to our study cohort (16,026). Therefore, our results indicated IMIDs, especially gout with chronic colitis having the colchicine use are associated with the lower risk of colorectal cancer. These speculations could infer to future research for relationship between the colchicine use in chronic colitis with gout among the IMIDs cohort.

### Strengths

This is the first general population study to investigate the effect of colchicine on the development of cancer in patients with the IMIDs-chronic colitis. The time-dependent and propensity score matching for avoiding the bias. Furthermore, in Taiwan, diagnoses of the cancer and the IMIDs follow strict guidelines. The role of colchicine in NLRP3 inflammasome activity has been examined in a Taiwanese’s study [54]. The diagnosis, treatment, or follow-up of these groups of patients drawn from the NHIRD were based on previous experiments. The strict policy contributing to our result would be representative of the real world in Taiwan. However, the result in our study warrants future research into the relationship between cancer, and the IMIDs-chronic colitis.

### Limitations

This study had a few limitations. First, the NHIRD provides no detailed information on patients regarding factors such as their lifestyle, body mass index (or obesity), habits (e.g., smoking, alcoholic drinking), physical activity, socioeconomic status, or family history; all of which are possible confounding factors in this study. We used alcohol-related diseases as a proxy for drinking, tobacco use and COPD for smoking, gout, hypertension and hyperlipidemia for exercise and life style [55, 56]. Yet, there are gaps between the proxy and real world. Second, the diagnosis of IMIDs in Taiwan is based on clinical history, imaging, laboratory data and pathology. The coding of the IMIDs in the NHIRD was established according to these strict standard [55, 56]. Notably, the registries in the NHI claims are primarily used for administrative billing and are not verified for scientific purposes. Third, recording of cancer diagnoses and survival estimates based on these diagnosis codes in the NHI database are generally consistent with the National Cancer Registry (NCR) [57]. However, lack of individual laboratory data, imaging finding, and pathologic result (such as cancer staging) in the NHIRD may be the other study limitation. Fourth, the retrospective study is usually lower evidence than the randomized controlled trials because a retrospective study is subject to have many unknown or uncontrolled confounding factors. Fifth, the incidence of some specific cancers was relatively low, thus limiting the power of our study. Sixth, as mention before, detection bias is present when the exposure (e.g., newly diagnosed patients with the IMIDs leads to higher detection of the outcome of interest (cancer) due to the increased frequency of clinic visits, which also results in an overestimation of risk-cancer. Thus, including a lag period, such as starting follow-up after 1 year of the initiation of a drug in this study, simultaneously considers a latency period while also minimizing protopathic and detection bias. However, colchicine use may just indicate developments around an imminent cancer diagnosis; these two bias were another limitation in this study.

## Conclusion

This study implied colchicine is associated with lower risk of all cancer and colorectal cancer formation in patients with IMIDs. However, age, sex comorbidities and detection bias may modulate the aHRs for brain and colorectal cancer in the colchicine users among the IMIDs.

### Supplementary Information

**Additional file 1: Figure S1.** Flow chart of selection of patients.

**Additional file 2: Table S1.** Full name of ICD-9CM with immune-mediated inflammatory diseases.

**Additional file 3: Table S2.** Incidence and HRs of all cancer in the colchicine cohorts compared with those in the non-colchicine cohorts by Cox proportional hazard models with time-dependent exposure covariates.

**Additional file 4: Table S3.** Incidence and HRs of cancer in the colchicine cohorts compared with those in the non-colchicine cohorts by Cox proportional hazard models with time-dependent exposure covariates in immune-mediated inflammatory diseases -chronic colitis in young adult

**Additional file 5: Table S4a.** The crude HR and aHR for the individual comorbidity in the colchicine use and non-colchicine use among the immune-related cohort by Cox proportional hazard model with time-dependent covariates in propensity-score-matched cohorts.

**Additional file 6: Table S4b.** The crude HR and aHR for the individual medication in the colchicine use and non-colchicine use among the immune-related cohort by Cox proportional hazard model with time-dependent covariates in propensity-score-matched cohorts.

## Data Availability

The dataset used in this study is held by the Taiwan Ministry of Health and Welfare (MOHW). The Ministry of Health and Welfare must approve our application to access this data. Any researcher interested in accessing this dataset can submit an application form to the Ministry of Health and Welfare requesting access. Please contact the staff of MOHW (Email: stcarolwu@mohw.gov.tw) for further assistance. Taiwan Ministry of Health and Welfare Address: No.488, Sec. 6, Zhongxiao E. Rd., Nangang Dist., Taipei City 115, Taiwan (R.O.C.). Phone: + 886-2-8590-6848. All relevant data are within the paper.

## Abbreviations

aHRs: Adjusted hazard ratios
CIs: Confidence intervals
NLRP3: NOD-like receptor protein 3
ICD-9-CM: International Classification of Diseases, Ninth revision, Clinical Modification
NHIRD: National Health Insurance Research Database
CAD: Coronary artery disease
COPD: Chronic obstructive pulmonary disease
ATC: Anatomical Therapeutic Chemical
PH: Proportional hazards
OSs: Oral steroids
NSAIDs: Non-steroidal anti-inflammatory drugs
IMIDs: Immune-mediated inflammatory diseases
