# Supplementary figures and images for: The effect of colchicine on cancer risk in patients with immune-mediated inflammatory diseases: a time-dependent study based on the Taiwan’s National Health Insurance Research Database
Source: Eur J Med Res. 2024 Apr 22;29:245. doi: 10.1186/s40001-024-01836-1 (PMC11034118; doi:10.1186/s40001-024-01836-1)

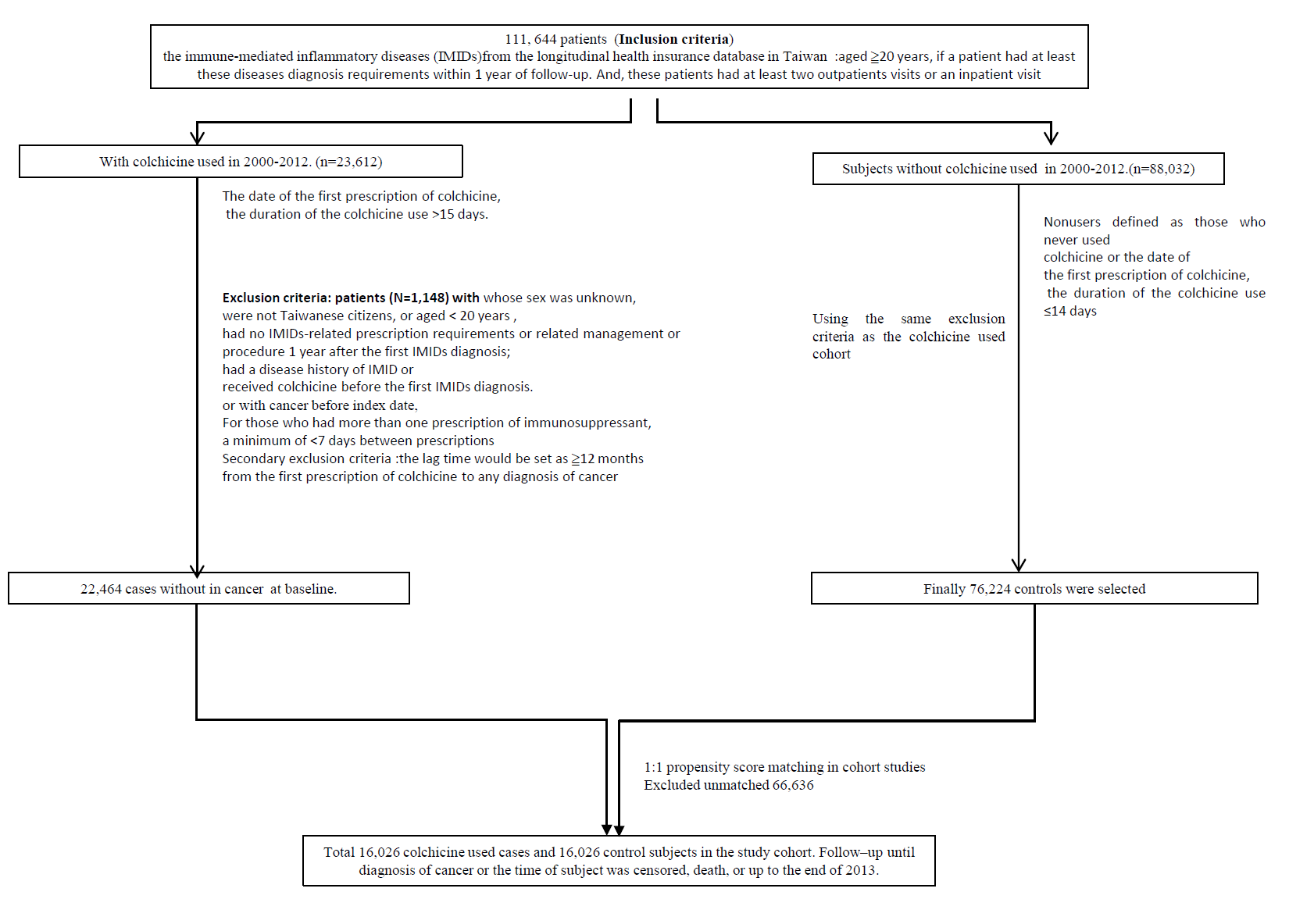

Supplement: Supplementary file 1 — Additional file 1: Figure S1. Flow chart of selection of patients. [file 40001_2024_1836_MOESM1_ESM.tif]
