# Supplementary material for: The effect of colchicine on cancer risk in patients with immune-mediated inflammatory diseases: a time-dependent study based on the Taiwan’s National Health Insurance Research Database
Source: Eur J Med Res. 2024 Apr 22;29:245. doi: 10.1186/s40001-024-01836-1 (PMC11034118; doi:10.1186/s40001-024-01836-1)
Supplement: Supplementary file 2 — Additional file 2: Table S1. Full name of ICD-9CM with immune-mediated inflammatory diseases. [file 40001_2024_1836_MOESM2_ESM.docx]

**Table S1.** Full name of ICD-9CM with immune-mediated inflammatory diseases.

| **Immune-mediated inflammatory diseases –related to colchicine use** | |
| --- | --- |
| **ICD-9CM (the rate of colchicine use)** | **FULL NAMES** |
| 274 (75%) | Gout |
| 712 (5%) | crystal arthropathies |
| Immune-mediated inflammatory diseases with secondary gout or in relation to gout or coexistence with gout or indication of colchicine use | |
| 135 (1.0 %) | Sarcoidosis |
| 136.1 (1.5%) | Behcet's syndrome |
| 279.49 (5.6%) | autoimmune disease |
| 518.3 (0.5%) | chronic idiopathic or spontaneous urticarial skin diseases |
| 287.0 (0.8%) | allergic purpura |
| 696.0, 696.1, 696.8 (2.5%) | Psoriasis |
| 420 ,429.4 (1.5%) | pericarditis, postcoricardiotomy syndrome (autoimmune inflammatory reaction within pleural and pericardial space) |
| 710.0,  710.1, 710.2, 710.3, 710.4. 714.0, 720 (5.4%) | diffuse diseases of connective tissue  systemic sclerosis Sjögren’s disease  dermatomyositis polymyositis  rheumatoid arthritis ankylosing spondylitis and other inflammatory spondylopathie |
| Chronic Colitis (1.2%) | |
| 555.0-555.1  555.9  556  45355, 45378, 45379, 45380, 45381, 45382, 45383, 45388, 45384, 45385, 45386, 45387, 45389, 45391, 45392, 45390, 45393, 45398, 45399 | Crohn's disease (similar symptom of the colorectal cancer)  ulcerative colitis  Current Procedural Terminology (CPT) codes |
| **Comorbidities** | |
| **ICD-9 CM** | **Full name** |
| Alcohol related disease | 291, 303, 571.0, 571.1, 571.2, 571.3, 790.3, and V11.3 |
| 410-414 | coronary artery disease |
| 250.0 | diabetes |
| 401-405 | hypertension |
| 272 | hyperlipidemia |
| 490-492, 496 | chronic obstructive pulmonary disease |
| 430-438 | stroke |
| 305.1, 305.11, 305.12, and 305.13  649.01 | tobacco use |
| 296.2, 296.3, 300.4, 311 | depression |
| 585 | chronic kidney disease |
| **ATC codes** | |
| A005225100, A0217541G0, A022077100, A022077100, A022534100, A030396100, A0303961G0, A041316100, A0413161G0, A046680100, A048749100, A0487491G0, A054643100, AC54643100, B022246100, N006271100, and N0062711G0) | colchicine |
| [R05](https://de.wikipedia.org/wiki/Kategorie:ATC-R05)[CB01](https://www.whocc.no/atc_ddd_index/?code=R05CB01), [V03](https://de.wikipedia.org/wiki/Kategorie:ATC-V03)[AB23](https://www.whocc.no/atc_ddd_index/?code=V03AB23), and [S01](https://de.wikipedia.org/wiki/Kategorie:ATC-S01)[XA08](https://www.whocc.no/atc_ddd_index/?code=S01XA08) | acetylcysteine |
| D07AC15, R01AD05, H02AB02, D07AC17, S01BA02, H02AB04, H02AB06, H02AB08, and S01BA02 | oral steroids, anti-inflammatory drugs |
| M01A | NSAIDs anti-inflammatory drugs |
| B01AC06 | acetylsalicylic acid anti-inflammatory drugs |
| N07BA01 N07BA03 | Smoking cessation related drugs (NRT, varenicline) |
|  | Immunosuppprasants |
| L04AX01 | azathioprine immunosuppressant drugs |
| A07EC01 | sulfasalazine immunosuppressant drugs |
| L01AA01 | cyclophosphamide immunosuppressant drugs |
| L01BA01 | methotrexate immunosuppressant drugs |
| P01BA02 | hydroxychloroquine immunosuppressant drugs |
| L04AD01 | cyclosporine immunosuppressant drugs |
| **procedure payment codes of NHIRD** | |
| **CODES** | **FULL NAMES** |
| 28017C  49014C  49025C  49027C | Colonoscopy  Colonoscopic polypectomy  Colonoscopy, with removal of foreign body |
| 28013C-28016C | Panendocopy- related |
| 28002C  28003C  28004C | Nasopharyngoscopy-related  Nasopharyngoscopy  Sinoscopoy  Flexible laryngoscopy |
| 33066B | Tomography (each) |
| 33070B-33072B | Brain CT- related Computed Tomography |
| 33084B-33085B | Brain MRI –related Magnetic resonance imaging |
| 19012C  20013B | ECHO- related |
| 79416C | Transrectal ultrasound guided prostate biopsy |
| 79401-79415 | Prostate –related |
| E1006C E1007C E1008C E1009D E1010D E1011C E1012C  E1013B E1014B  E1015B E1016B E1017A E1018A E1019A  E1020A E1002C  E1003C E1004C E1005C  B0220903EF B022089396  B0220883EG A0386463EH A0386463EH  A0386463EK B022538129  B019312100 B019313100 B023941100 E1006C  E1002C E1009D  B0220903EF | Medication for Tobacco dependence -related |
