# Supplementary material for: The effect of colchicine on cancer risk in patients with immune-mediated inflammatory diseases: a time-dependent study based on the Taiwan’s National Health Insurance Research Database
Source: Eur J Med Res. 2024 Apr 22;29:245. doi: 10.1186/s40001-024-01836-1 (PMC11034118; doi:10.1186/s40001-024-01836-1)
Supplement: Supplementary file 4 — Additional file 4: Table S3. Incidence and HRs of cancer in the colchicine cohorts compared with those in the non-colchicine cohorts by Cox proportional hazard models with time-dependent exposure covariates in immune-mediated inflammatory diseases -chronic colitis in young adult [file 40001_2024_1836_MOESM4_ESM.docx]

| Table S3. Incidence and HRs of cancer in the colchicine cohorts compared with those in the non-colchicine cohorts by Cox proportional hazard models with time-dependent exposure covariates in immune-mediated inflammatory diseases -chronic colitis in young adult | | | | |
| --- | --- | --- | --- | --- |
|  | **No Matched** | | **Propensity Score Matched** | |
|  | **Colchicine** | | **Colchicine** | |
|  | **No** | **Yes** | **No** | **Yes** |
|  | **(N=854)** | **(N=210)** | **(N=182)** | **(N=182)** |
| **Person-years** | 64078 | 2690 | 1248 | 1217 |
| **Follow-up time (y), Median±(IQR)** | 7.22(4.00-10.5) | 7.76(4.42-10.9) | 7.80(4.51-11.1) | 7.76(4.42, 11.0) |
| **Cancer** |  |  |  |  |
| Event | 53 | 15 | 14 | 11 |
| Rate ^#^ | 7.01 | 6.51 | 7.03 | 6.02 |
| **Crude HR (95% CI)** | 1(Reference) | 0.92(0.53,0.96)* | 1(Reference) | 0.86(0.61, 0.98)* |
| **Adjusted HR†(95% CI)** | 1(Reference) | 0.90(0.50,0.97)* | 1(Reference) | 0.81 (0.53, 0.99)* |

Rate#, incidence rate, per 1000 person-years; Crude HR, relative; Adjusted HR†: multivariable analysis including age, sex, comorbidities and medications

**P* <0 .05
