# Supplementary material for: The effect of colchicine on cancer risk in patients with immune-mediated inflammatory diseases: a time-dependent study based on the Taiwan’s National Health Insurance Research Database
Source: Eur J Med Res. 2024 Apr 22;29:245. doi: 10.1186/s40001-024-01836-1 (PMC11034118; doi:10.1186/s40001-024-01836-1)
Supplement: Supplementary file 5 — Additional file 5: Table S4a. The crude HR and aHR for the individual comorbidity in the colchicine use and non-colchicine use among the immune-related cohort by Cox proportional hazard model with time-dependent covariates in propensity-score-matched cohorts. [file 40001_2024_1836_MOESM5_ESM.docx]

Table S4a. The crude HR and aHR for the individual comorbidity in the colchicine use and non-colchicine use among the immune-related cohort by Cox proportional hazard model with time-dependent covariates in propensity-score-matched cohorts.

| **Comorbidities** | **Comparision of Colchicine user with non-colchicine user** | |
| --- | --- | --- |
|  | **Crude HR (95 % CI)** | **Adjusted HR† (95 % CI)** |
| **Alcohol-related illness** |  |  |
| No | 1(Reference) | 1(Reference) |
| Yes | 1.27(1.02, 3.55) * | 1.47(1.01,1.85) * |
| **Coronary artery disease** |  |  |
| No | 1(Reference) | 1(Reference) |
| Yes | 1.87(0.43, 3.55) | 1.91(0.51, 2.45) |
| **Diabetes** |  |  |
| No | 1(Reference) | 1(Reference) |
| Yes | 1.37(1.13, 2.14) * | 1.38(1.03, 3.55) * |
| **Hypertension** |  |  |
| No | 1(Reference) | 1(Reference) |
| Yes | 1.67(0.53, 4.25) | 1.97(0.85, 2.65) |
| **Hyperlipidemia** |  |  |
| No | 1(Reference) | 1(Reference) |
| Yes | 1.51(1.01, 2.55) * | 1.54 (1.03, 2.75) * |
| **COPD** |  |  |
| No | 1(Reference) | 1(Reference) |
| Yes | 2.97(2.53, 3.95) ** | 2.99(2.63, 4.55) ** |
| **Stroke** |  |  |
| No | 1(Reference) | 1(Reference) |
| Yes | 1.07(0.93, 3.20) | 1.23(0.83, 2.65) |
| **Depression** |  |  |
| No | 1(Reference) | 1(Reference) |
| Yes | 2.21(1.53, 2.57) ** | 2.25(1.48, 2.98) ** |
| **Tobacco use** |  |  |
| No | 1(Reference) | 1(Reference) |
| Yes | 2.05(1.03, 3.55) ** | 2.67(1.02, 3.99) ** |
| **Chronic kidney disease** |  |  |
| No | 1(Reference) | 1(Reference) |
| Yes | 1.33(1.23, 2.89) * | 1.77(1.13, 2.88) * |

Crude HR, relative hazard ratio;

†Adjusting for age, sex, comorbidities and medications;

*p<0.05, **p<0.01
