# Supplementary material for: The effect of colchicine on cancer risk in patients with immune-mediated inflammatory diseases: a time-dependent study based on the Taiwan’s National Health Insurance Research Database
Source: Eur J Med Res. 2024 Apr 22;29:245. doi: 10.1186/s40001-024-01836-1 (PMC11034118; doi:10.1186/s40001-024-01836-1)
Supplement: Supplementary file 6 — Additional file 6: Table S4b. The crude HR and aHR for the individual medication in the colchicine use and non-colchicine use among the immune-related cohort by Cox proportional hazard model with time-dependent covariates in propensity-score-matched cohorts. [file 40001_2024_1836_MOESM6_ESM.docx]

Table S4b. The crude HR and aHR for the individual medication in the colchicine use and non-colchicine use among the immune-related cohort by Cox proportional hazard model with time-dependent covariates in propensity-score-matched cohorts.

| **Medications** | **Colchicine use with non-colchicine use** | |
| --- | --- | --- |
|  | **Crude HR (95 % CI)** | **Adjusted HR† (95 % CI)** |
| **Acetylcysteine** |  |  |
| No | 1(Reference) | 1(Reference) |
| Yes | 1.13(0.25, 2.45) | 1.47(0.58,1.98) |
| **Smoking cessation-related** |  |  |
| No | 1(Reference) | 1(Reference) |
| Yes | 0.87(0.43, 0.99) * | 0.91(0.51, 0.98) * |
| **Anti-inflammatory** |  |  |
| No | 1(Reference) | 1(Reference) |
| Yes | 0.85(0.11, 0.91) * | 0.78(0.55, 0.94) * |
| **Immunosuppressant drugs** |  |  |
| No | 1(Reference) | 1(Reference) |
| Yes | 1.17(1.11, 2.10) * | 1.28(1.01, 2.25) * |

Crude HR, relative hazard ratio;

†Adjusting for age, sex, comorbidities and medications;

*p<0.05, **p<0.01
